# The neuroendocrine stress response compensates for suppression of insulin secretion by volatile anesthetic agents: An observational study

**DOI:** 10.14814/phy2.15603

**Published:** 2023-02-17

**Authors:** William G. Tharp, Max W. Breidenstein, Alexander F. Friend, S. Patrick Bender, Daniel Raftery

**Affiliations:** ^1^ Department of Anesthesiology University of Vermont Medical Center Burlington Vermont USA; ^2^ Department of Anesthesiology and Pain Medicine University of Washington Seattle Washington USA

## Abstract

Alterations in perioperative metabolic function, particularly hyperglycemia, are associated with increased post‐operative complications, even in patients without preexisting metabolic abnormalities. Anesthetic medications and the neuroendocrine stress response to surgery may both contribute to altered energy metabolism through impaired glucose and insulin homeostasis but the discrete pathways involved are unclear. Prior human studies, though informative, have been limited by analytic sensitivity or technique, preventing resolution of underlying mechanisms. We hypothesized that general anesthesia with a volatile agent would suppress basal insulin secretion without altering hepatic insulin extraction, and that surgical stress would promote hyperglycemia through gluconeogenesis, lipid oxidation, and insulin resistance. In order to address these hypotheses, we conducted an observational study of subjects undergoing multi‐level lumbar surgery with an inhaled anesthetic agent. We measured circulating glucose, insulin, c‐peptide, and cortisol frequently throughout the perioperative period and analyzed the circulating metabolome in a subset of these samples. We found volatile anesthetic agents suppress basal insulin secretion and uncouple glucose‐stimulated insulin secretion. Following surgical stimulus, this inhibition disappeared and there was gluconeogenesis with selective amino acid metabolism. No robust evidence of lipid metabolism or insulin resistance was observed. These results show that volatile anesthetic agents suppress basal insulin secretion, which results in reduced glucose metabolism. The neuroendocrine stress response to surgery ameliorates the inhibitory effect of the volatile agent on insulin secretion and glucose metabolism, promoting catabolic gluconeogenesis. A better understanding of the complex metabolic interaction between anesthetic medications and surgical stress is needed to inform design of clinical pathways aimed at improving perioperative metabolic function.

## Graphical abstract

Metabolomic profiling during surgery with a volatile anesthetic agent. Insulin secretion is suppressed by volatile anesthetic agents resulting in decreased glucose metabolism. The neuroendocrine stress response to surgery ameliorates the impairment of glucose‐insulin homeostasis.

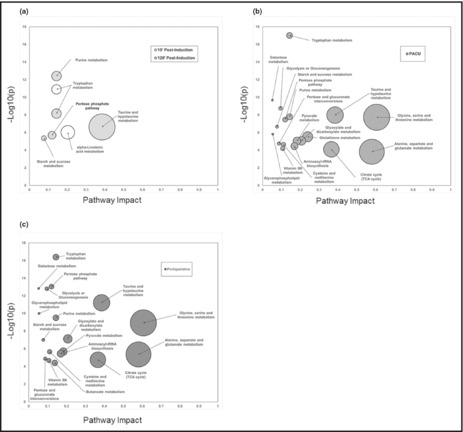

## 1 INTRODUCTION

Energy metabolism becomes greatly deranged in the perioperative setting. Altered metabolic function, especially hyperglycemia, has been associated with increased postoperative morbidity and mortality, even in patients without diabetes or metabolic syndrome (Abdelmalak et al., 2014; Akhtar et al., 2010; Bagry et al., 2008; Noordzij et al., 2007). Perioperative contributions to altered metabolic function include preoperative fasting, effects of anesthetic medications, and activation of the neuroendocrine stress response by the surgical procedure. The effects of metabolic dysregulation are deleterious. Poor glycemic control in patients with type 2 diabetes mellitus is associated with impaired wound healing and increased mortality following surgery (Noordzij et al., 2007; Tsourdi et al., 2013). Acute changes in glucose and insulin levels can alter platelet function and induce inflammation (Konieczynska et al., 2013; McGovern et al., 2012; Spectre et al., 2012). In attempting to prevent these metabolic derangements, enhanced recovery pathways often incorporate protocols for perioperative glucose control, preoperative carbohydrate loading, and rapid return to postoperative oral nutrition (Memtsoudis et al., 2019). While there is an emerging recognition that perioperative metabolic function may significantly impact surgical course and outcomes, the contributing mechanisms are unclear.

The neuroendocrine stress response to injury is characterized by increased sympathetic nervous system activity, elaboration of catecholamines, and cortisol release (Carli & Schricker, 2000; Miller & O'Callaghan, 2002; Schricker et al., 2001; Schricker et al., 2005). This response reduces sensitivity to insulin, promotes hyperglycemia, and stimulates proteolysis. In the perioperative period, the stress response to surgical intervention may be altered by anesthetic medications. Volatile anesthetic agents appear to have a direct effect on insulin secretion in vitro, in canine and porcine models, and possibly in humans (Desborough et al., 1993; Gingerich et al., 1974; Horber et al., 1990; Saho et al., 1997; Tanaka et al., 2009; Vore et al., 2001). However, our understanding of glucose and insulin homeostasis during anesthesia and surgery is limited by a lack of simple, high‐resolution, observational data in humans (Diltoer & Camu, 1988; Iwasaka et al., 1996; Oyama, 1973; Tanaka et al., 2005; Tsubo et al., 1990). The effects of volatile anesthetics on basal insulin secretion and hepatic insulin extraction, an indicator of insulin resistance, are unknown. Beyond glucose and insulin homeostasis, only a few studies have attempted to examine the natural perioperative course of protein or lipid metabolism (Carli et al., 2005; Schricker et al., 2001). These studies demonstrated that pathways beyond glucose homeostasis are likely critical components of perioperative metabolism, but were limited by available analytic techniques and complex stable isotope labeling methods.

We hypothesized that general anesthesia with a volatile agent would suppress basal insulin secretion without altering hepatic extraction and that subsequent surgical stress would promote hyperglycemia through protein catabolism, lipid oxidation, and uncoupled glucose‐insulin homeostasis. To address these hypotheses, we conducted an observational study of perioperative metabolic function in patients undergoing multi‐level spine surgery with an inhaled anesthetic agent. We examined glucose and insulin homeostasis using sensitive analytic methods and we used targeted metabolomics analyses to make a high‐resolution assessment of key perioperative metabolic pathways.

## 2 METHODS

### 2.1 Clinical study design

We conducted an observational study of patients undergoing multi‐level spine surgery with the approval of the University of Vermont Institutional Review Board (M14‐291; NCT04396964). All subjects gave written informed consent prior to participation. Subjects were eligible if they were >18 years old without acute or chronic health conditions including cardiac, pulmonary, hepatic, renal, auto‐immune, or hematological disease. Obese subjects with body mass index >40 kg/m^2^ were excluded. Subjects with Type 2 diabetes were eligible if they were well‐controlled (HbA1c < 7.5%) and treated with diet and exercise, metformin, or insulin. Subjects were excluded if taking other hypoglycemic agents, weight loss medication, or other medications affecting glucose homeostasis. Enrollment lasted from 2014–2016.

**FIGURE 1:**
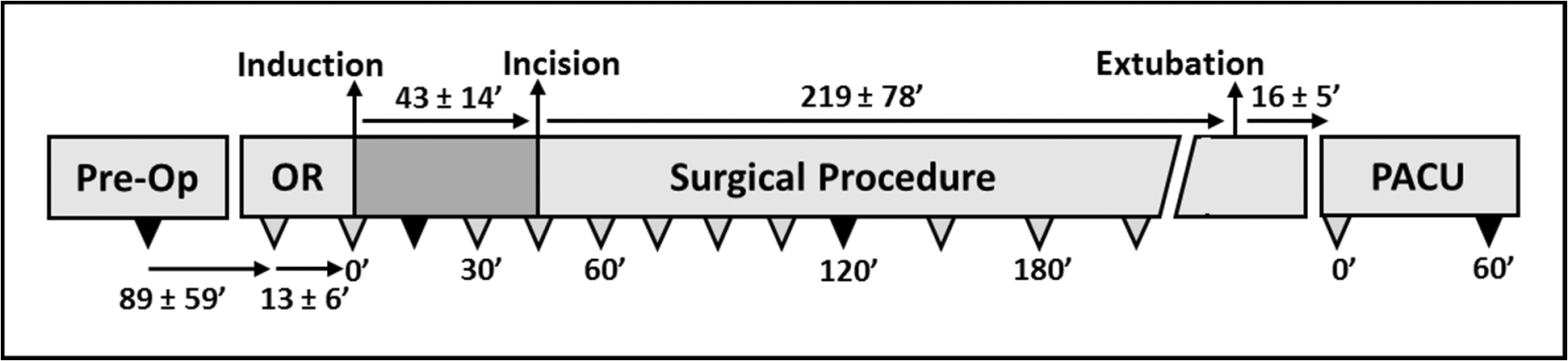
Perioperative events and blood collection schematic. Triangles represent blood sampling. Black triangles denote samples for metabolomic analyses. A sample was drawn in pre‐operative hold (Pre‐Op) and another upon arrival to the operating room (OR). A sample was drawn immediately following induction, every 15 min for 2 h, then every 30 min until extubation. Finally, a sample was drawn on arrival to the post‐anesthesia care unit (PACU) and again 60 min later. Average time (minutes ± SD) until the next event is noted on the schematic.

Subjects presented to preoperative hold after an overnight fast and had a dedicated peripheral intravenous line placed for blood sampling. No medications were administered through this line. Baseline samples were collected in preoperative hold and after transfer to the operating room. After the blood sampling in preoperative hold, patients received any medications associated with an enhanced recovery pathway. Following induction and intubation blood samples were collected at 15′ intervals for the initial 2 h of each case, then at 30′ intervals for the remainder of the case until emergence and extubation. A blood sample was collected on arrival to the post‐anesthesia care unit and again 1 h later (Figure 1). Blood samples were collected on ice and aliquoted into potassium oxalate, potassium EDTA, and serum‐separating tubes. Samples were processed immediately and stored at −80°C until analyzed. Demographic and anthropomorphic data were collected from the medical record. Intraoperative data were collected from the anesthesia record. Hemodynamic data were aligned to the time of intubation and underwent a 5‐min average smoothing.

### 2.2 Hormone measurements

Glucose was measured using the glucose oxidase method on a dedicated platform (Yellow Spring International). Insulin and cortisol were measured by immunoassay (Alpco) with 3.8% and 4.2% average coefficient of variation (CoV), respectively. C‐peptide was measured by electrochemiluminescent immunoassay (MesoScale Discovery) with 4.3% CoV.

### 2.3 Calculations

Fold change calculation were made for glucose, insulin, c‐peptide, and cortisol using the initial pre‐operative sample as the reference. Insulin‐to‐glucose ratio was calculated in pM/mM. Hepatic extraction was estimated as 1—(insulin [pM]/c‐peptide [pM]). Insulin secretion rates were calculated from c‐peptide levels by population deconvolution of a two‐compartment model (Hovorka et al., 1996).

### 2.4 Metabolomics

Metabolites were measured in a subset of plasma samples by targeted LC–MS/MS at the Northwest Metabolomics Research Center at the University of Washington. Up to 107 metabolites were detected per samples alongside 33 labeled internal standards. Quality control samples were run every 10 samples using both instrument standards and pooled plasma samples with CoV of 4.7% and 4.6%, respectively. Absolute quantification was performed for 30 metabolites using labeled standards.

### 2.5 Statistical analyses

For an anesthetic duration of 120 min or more a subject would have ≥13 blood samples (2 pre‐induction, ≥9 under anesthesia, and 2 in PACU). Using a repeated measures model with 10 measurements we originally estimated 14 subjects would be needed to measure a 25% change in basal glucose or insulin levels at 80% power and an alpha of 0.05. Preoperative and intraoperative data were subsequently analyzed with mixed effects linear modeling using time as a fixed effect and subject as a random effect; this approach accommodated missing data points. Time series data are presented censored after 270 min in the manuscript as only four subjects had anesthetic duration greater than 300 min.

Metabolite data were analyzed using the online implementation of Metaboanalyst 4.0 (www.metaboanalyst.ca) (Chong et al., 2019). Metabolites with <50% detection were excluded from the analyses and missing data were replaced with a value equal to half the minimum value in the original data. Sample data were auto‐scaled (mean‐centered and normalized to the standard deviation of the metabolite data) prior to analysis (Figure S2). Changes in metabolite concentration over time were assessed by one‐way ANOVA with the Benjamini–Hochberg false discovery rate (FDR) correction and post‐hoc Tukey honest significant difference test. Correlation of metabolite temporal patterns was assessed with Pearson coefficients and hierarchical clustering. Dimension reduction used partial least squares discriminant analysis (PLS‐DA) and variable importance in projection (VIP) measurement. Pathway analyses utilized the Kyoto Encyclopedia of Genes and Genomes (KEGG) *Homo sapiens* reference library under the global test for enrichment and relative‐betweenness centrality for topology. Metabolite enrichment analyses used pathway‐associated, predicted, and disease‐associated metabolite reference libraries.

## 3 RESULTS

### 3.1 Subject, anesthetic, and surgical characteristics

We enrolled 16 subjects undergoing multi‐level spine surgeries with an inhaled anesthetic. Demographic, clinical, and perioperative data are shown in Table 1. Fourteen subjects had multi‐level lumbar fusions, two had a multi‐level thoracic fusion without opening of the pleura. Aside from the duration the procedures, temporal events around administration of volatile anesthetic agents were similar (Figure 1). Intervals between blood samples around intubation and extubation did not vary widely. The time from intubation to incision averaged 43 ± 14 min. Nine subjects had intubation times before 9:30, and seven had intubation times between 11:00 and 14:00 h.

**TABLE 1:** Subject and procedure characteristics.

Demographics	
Sex (M/F)	11/5
ASA (1/2/3)	3/11/2
Age (years)	59 ± 11
Weight (kg)	84 ± 17
BMI (kg/m^2^)	28 ± 3
Obesity (BMI ≥ 30 kg/m^2^)	5
Type 2 diabetes (HbA1c > 6.5%)	3

Procedure characteristics	
Location (lumbar/thoracic)	14/2
Anesthesia duration (min)	276 ± 90
Surgical duration (min)	218 ± 78
Crystalloid administered (L)	3.0 ± 1.4
Estimated blood loss (mL)	590 ± 500
Blood transfusion (Y/N)	3/13
Autologous/Cell salvage	2
Donor derived packed RBC	1

*Note*: All values either counts or mean ± standard deviation. Abbreviations: BMI, body mass index; HOMA‐IR, homeostatic model of insulin resistance; HbA1c, hemoglobin A1c; RBC, red blood cells.

As part of enhanced recovery pathway, 12 subjects received either pregabalin or gabapentin, oxycodone or morphine, and diazepam preoperatively; a subset also received acetaminophen, celecoxib, and dexamethasone (Table 2). Induction medications were similar across the cohort and comprised of a benzodiazepine, propofol, lidocaine, an opioid, ketamine, and an aminosteroid neuromuscular blocker. All subjects were maintained on an inhaled volatile agent (11 sevoflurane, four isoflurane, one desflurane).

**TABLE 2:** Perioperative medications.

Pre‐operative medications	# subjects receiving	Dose
Acetaminophen	6	1000 mg
Celecoxib	4	200 mg
Pregabalin/Gabapentin	7/5	150 mg/600 mg
Diazepam	12	10 mg
Oxycodone IR/Morphine IR	8/4	10–20 mg/15–30 mg
Dexamethasone (oral)	4	8 mg
Induction/Pre‐incision medications		
Midazolam (IV)	14	3 ± 1 mg
Morphine equivalents (IV)	15	13 ± 9 mg
Lidocaine (IV)	14	63 ± 17 mg
Ketamine (IV)	15	29 ± 11 mg
Propofol (IV)	16	194 ± 47 mg
Rocuronium equivalents (IV)	16	51 ± 6 mg
Dexamethasone (IV)	7	7 ± 3 mg
Volatile anesthetic agent		
Sevoflurane	11	
Isoflurane	4	
Desflurane	1	

*Note*: All values either counts or mean ± standard deviation.

Intravenous crystalloid administration averaged 2950 ± 1400 mL and estimated blood loss averaged 590 ± 500 mL. No subjects received colloids. Two subjects received autologous erythrocytes from intraoperative blood salvage during surgical closure (these did not contain glucose or preservatives). One subject received donated erythrocytes, containing glucose and preservatives, at 150 min after induction of anesthesia. Two subjects were fed in PACU prior to the final blood draw. Anesthetic and surgical duration varied widely (Figure S1). Time series data are censored after 270 min as only four subjects had anesthetic duration over 300 min.

Mean arterial pressures decreased from 88 ± 15 mmHg pre‐induction to 76 ± 13 mmHg 10 min after induction (*p* < 0.03) and remained in this range for the duration of the surgical case (Figure S1). Heart rates averaged 74 ± 10 bpm pre‐induction, did not change after intubation (77 ± 10 bpm, *p* > 0.1), and were similar to baseline until 230 min following intubation, after which average heart rates increased, becoming persistently higher in subjects with surgeries lasting more than 240 min (*p* < 0.05; Figure S1).

### 3.2 Perioperative circulating glucose, insulin, and cortisol concentrations

**FIGURE 2:**
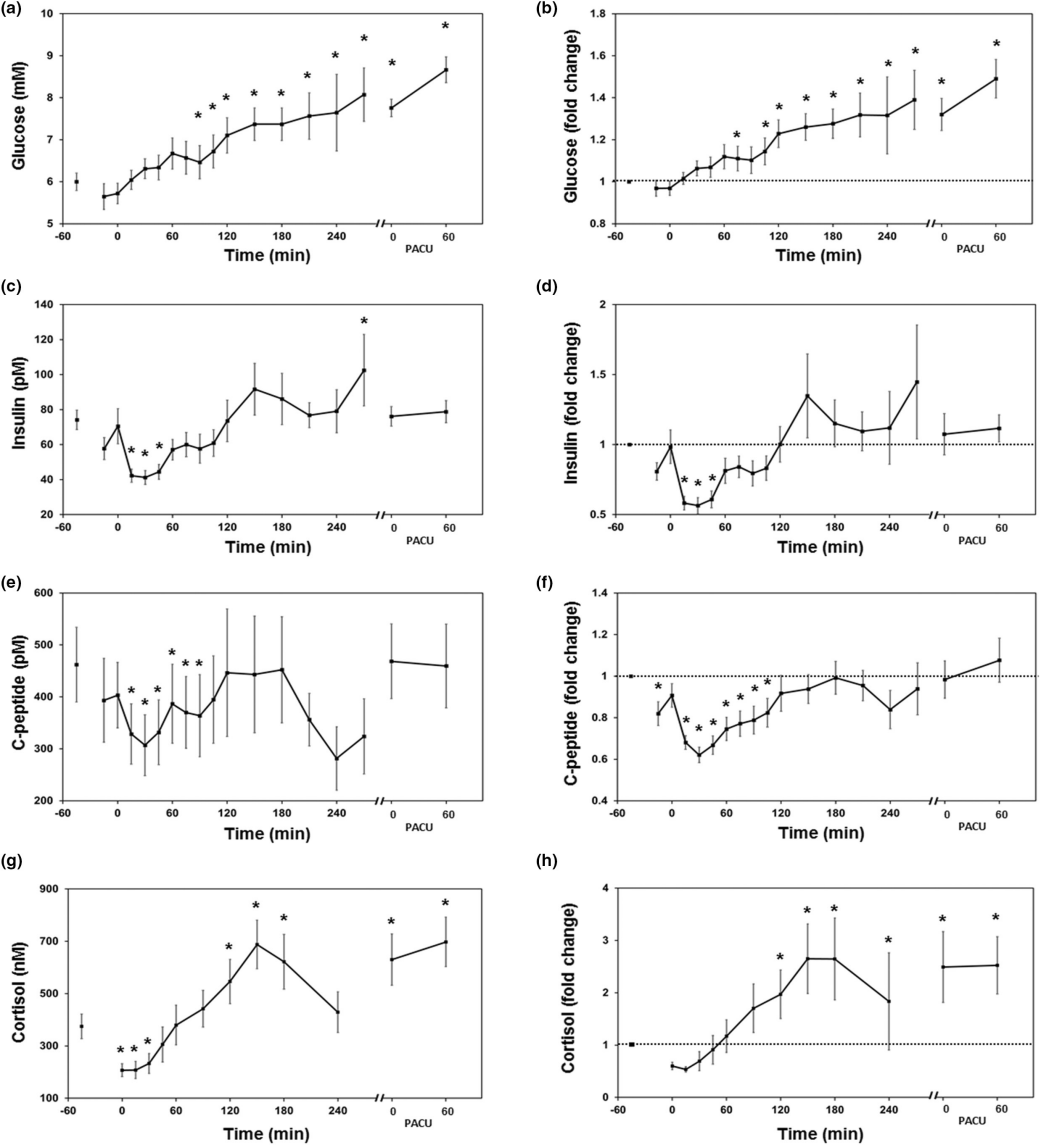
Perioperative glucose, insulin, and cortisol dynamics. Glucose concentrations (a) and fold change from baseline (b) over the perioperative period. Insulin concentrations (c) and fold change from baseline (d). Circulating c‐peptide levels (e) and fold change from baseline (f). Perioperative cortisol concentrations (g) and fold change from baseline (h). Data are presented as mean ± SEM and are censored between 270 min and arrival in the post‐anesthesia care unit (PACU) due to low subject number. Dashed line represents baseline. *p < 0.05 compared to first sample or baseline by linear mixed effects modeling.

Preoperative fasting glucose averaged 6.0 ± 0.2 mM, rose steadily following intubation, became elevated by more than 20% at 120 min, and averaged 7.8 ± 0.4 mM on arrival to PACU (Figure 2a,b). Peripheral insulin levels dropped by more than 40% from baseline averages of 74.2 ± 5.5 pm following intubation and remained depressed for 45 min post‐intubation (Figure 2c,d). To ensure this was not an effect of first‐pass hepatic insulin extraction we also measured c‐peptide concentrations. C‐peptide is produced by proteolytic cleavage of proinsulin, is secreted in equimolar amounts with insulin from the pancreatic beta cell, but is not removed from the portal circulation by the liver, allowing estimation of pancreatic insulin secretion. Circulating c‐peptide levels also fell rapidly after induction and remained significantly decreased up to 90 min after intubation (Figure 2e,f). We measured circulating cortisol concentrations to assess for initiation of the neuroendocrine stress response. Cortisol levels averaged 375 ± 47 nM pre‐operatively, decreased more than 40% in the first 30 min after intubation, and became elevated 120 min after intubation (Figure 2g,h). After extubation and arrival to PACU, cortisol concentrations were increased more than 50% compared to baseline. Analysis of cortisol and intubation time (before 9:30 vs. between 11:00 and 14:00) showed no differences in baseline cortisol levels (377 ± 73 vs. 377 ± 59 nM, *p* = 0.97).

**FIGURE 3:**
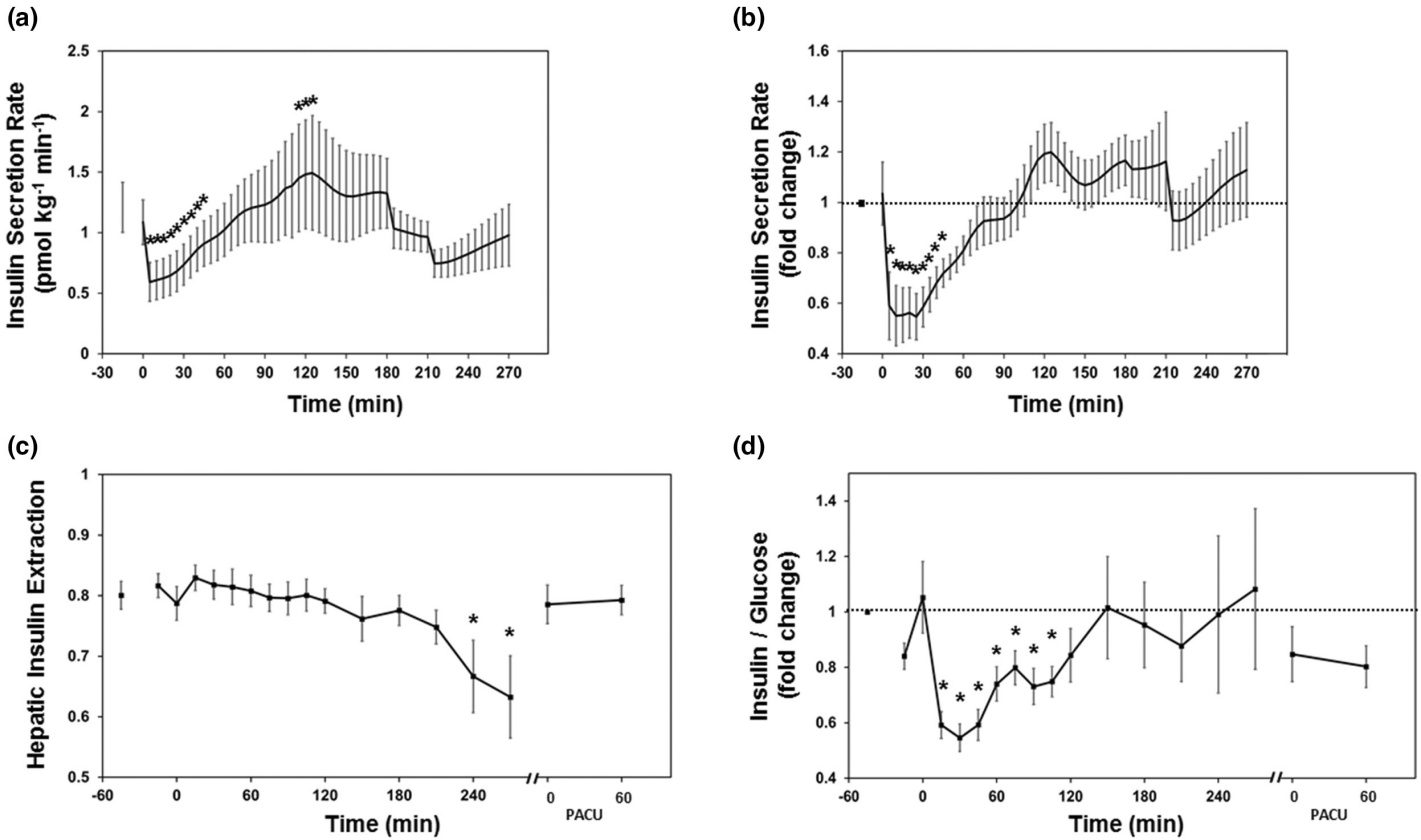
Alterations in perioperative insulin homeostasis. Insulin secretion rates (a) calculated from c‐peptide concentrations. Fold change insulin secretion rate from baseline (b; dashed line represents baseline). Hepatic extraction ratio derived from difference in circulating insulin and c‐peptide levels (c). Insulin‐to‐glucose ratios over the perioperative period (d). Data are presented as mean ± SEM. Insulin secretion rates were not calculated from post‐anesthesia care unit (PACU) data. Hepatic extraction and insulin‐to‐glucose ratio data are censored between 270 min and arrival in the PACU due to low subject number. Dashed line represents baseline. *p < 0.05 compared to first sample or baseline by linear mixed effects modeling.

Insulin secretion rates, derived from two‐compartment modeling of c‐peptide concentrations, were reduced following intubation and did not recover to baseline until 45 min post‐intubation (Figure 3a,b) (Hovorka et al., 1996). First‐pass hepatic insulin extraction, which typically ranges between 50%–80% in the fasting state, can be estimated using a simple insulin‐to‐c‐peptide molar ratio. Hepatic insulin extraction was estimated at 80 ± 9% at baseline, remained unchanged for 210 min, decreased to 63 ± 20% by 270 min, and returned to 79 ± 10% in PACU (Figure 3c). The insulin‐to‐glucose ratio can be used to estimate integrated β‐cell function and insulin sensitivity in the fasting state (Wallace & Matthews, 2002). Here we used this ratio to assess glucose and insulin homeostatic coupling during the perioperative period. The insulin‐to‐glucose ratio was decreased significantly for the first 105 min after intubation, returned to baseline values for the remainder of the surgical procedure, and was depressed following emergence (Figure 3d).

### 3.3 Metabolite changes in the perioperative period

**FIGURE 4:**
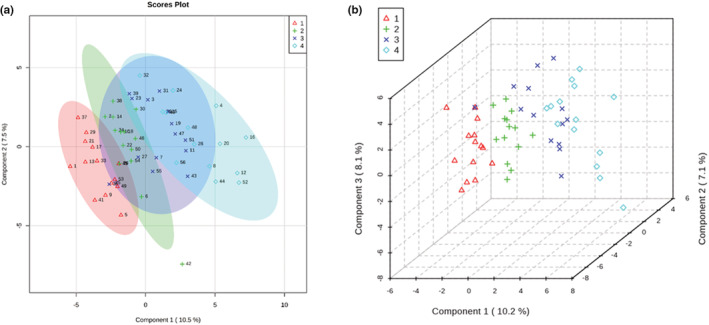
Perioperative alterations in the circulating metabolome. Score plots for the first two (a) and first three (b) components of the metabolite data from partial least squares discriminant analysis (PLS‐DA) show clear evolution over the perioperative period. Red triangles (1) are preoperative data, green pluses (2) are 15 min data, blue crosses (3) are 120 min data, and teal diamonds (4) are 60 min after arrival post‐anesthesia care unit (PACU) data. The shaded areas represent the 95% confidence regions for each time point. Percent variance explained by each component is noted in parentheses. Data labels are randomly assigned.

**FIGURE 5:**
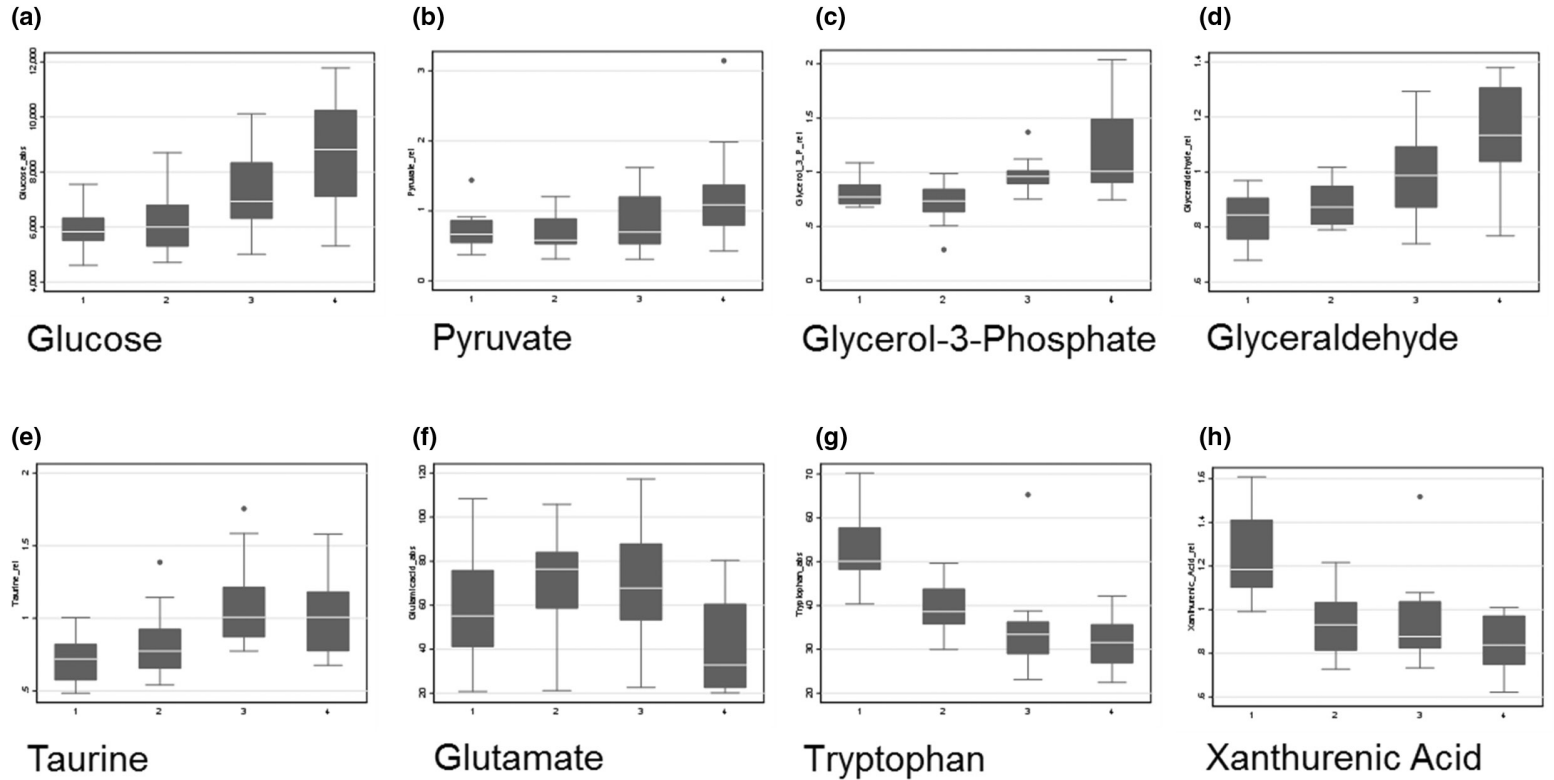
Perioperative carbohydrate and amino acid dynamics. Glucose (a), pyruvate (b), glycerol‐3‐phosphate (c), glyceraldehyde (d), and taurine (e) concentrations increase over the course of the perioperative period. Circulating glutamate concentrations are decreased in the post‐anesthesia care unit (f), while tryptophan (g) and xanthurenic acid (h) decreased steadily over the perioperative course. Time points are: 1—pre‐operative, 2–15 min after induction, 3–120 min after induction, and 4–60 min after arrival in post‐anesthesia care unit.

In order to assess the biochemical pathways involved in perioperative metabolic changes, we measured metabolites in plasma samples from 14 subjects at four time points: prior to induction, 15 and 120 min after intubation, and after 60 min in PACU (Figure 1). We excluded one subject from metabolite analysis due to shorter than anticipated surgical duration (<120 min) and one subject due to receiving a non‐autologous blood transfusion containing glucose and preservative solutions (this subject was also fed in PACU prior to the final blood draw). Sixteen metabolites changed significantly from baseline over the course of the perioperative period (after FDR correction with *p* < 0.05) (Table 3). We used PLS‐DA to assess changes in metabolite patterns over the perioperative period (Figure 4a,b). The first principal component accounted for 10.5% of the data variance and included glucose, pyruvate, lactate, glyceraldehyde, glycerol‐3‐phosphate, tryptophan, xanthurenic acid (8‐OH‐kynurenic acid), taurine, and D‐leucic acid. The second component accounted for 7.5% of the variance and included glutamine, phenylalanine, N‐acetylneuraminate, inositol, urate, glucoronate, adipic acid, orotate, alpha‐ketoglutarate, succinate, and fructose. Metabolites in the first two components were mainly involved in glycolysis, the tricarboxylic acid (TCA) cycle, metabolism of certain amino acids, and to a lesser extent lipid, nucleotide, and bile acid metabolism. VIP scores showed predominant alterations in substrates involved in glycolysis, metabolism of tryptophan, taurine, alanine, and leucine, and also in nucleotide and bile acid metabolism over the perioperative period (Table 4). One‐way ANOVA with FDR correction and post hoc testing showed substrates involved in carbohydrate metabolism and the tricarboxylic acid (TCA) cycle increased over the duration of the surgical period (Figure 5a–d). The neuroactive amino acid taurine increased following surgical intervention and remained elevated in PACU (Figure 5e). Glutamate was decreased in PACU compared to intraoperative levels (Figure 5f). Tryptophan and its metabolite, xanthurenic acid, decreased over the perioperative period (Figure 5g,h).

**TABLE 3:** Metabolite one‐way ANOVA data.

Metabolite	f value	*p* value	–log10(p)	FDR	Tukey's HSD
Tryptophan	20.51	0.00	8.18	0.00	2–1; 3–1; 4–1; 4–2
Xanthurenic acid	15.13	0.00	6.48	0.00	2–1; 3–1; 4–1
Glyceraldehyde	13.88	0.00	6.04	0.00	3–1; 4–1; 4–2; 4–3
Glycochenodeoxycholate	13.77	0.00	6.00	0.00	2–1; 3–1; 4–1
Uridine	10.84	0.00	4.91	0.00	2–1; 3–1; 4–1; 4–2; 4–3
Glucose	10.43	0.00	4.75	0.00	3–1; 4–1; 4–2; 4–3
Glycerol‐3‐phosphate	10.29	0.00	4.70	0.00	4–1; 3–2; 4–2; 4–3
13‐HODE	9.20	0.00	4.26	0.00	2–1; 3–2; 4–2
Xanthine	8.05	0.00	3.78	0.00	3–1; 4–1; 4–2
Taurine	7.50	0.00	3.54	0.00	3–1; 4–1; 3–2; 4–2
Linolenic acid	5.60	0.00	2.68	0.02	2–1; 3–2; 4–2
Propionate	5.38	0.00	2.58	0.02	4–1; 4–2; 4–3
Oxalic acid	5.11	0.00	2.45	0.03	3–1; 4–1; 4–2
D‐Leucic acid	4.64	0.01	2.22	0.05	4–1; 4–2
Glutamate	4.58	0.01	2.19	0.05	4–2; 4–3
Pyruvate	4.50	0.01	2.15	0.05	4–1; 4–2; 4–3
Aminolevulinate	4.33	0.01	2.07	0.05	3–1; 4–3

*Note*: The f‐value and unadjusted p‐value are from one‐way ANOVA of metabolite over time. Post hoc pairwise Tukey's test of the time points adjusted for multiple comparisons are shown in column 6. Time points are: 1—pre‐operative, 2–15 min after induction, 3–120 min after induction, and 4–60 min after arrival in post‐anesthesia care unit. Abbreviations: FDR, false detection rate; HSD, honest significant difference.

**TABLE 4:** Metabolite variable importance in projection component scores.

	Component				
Metabolite	1	2	3	4	5
Tryptophan	2.66	2.56	2.49	2.45	2.40
Glyceraldehyde	2.49	2.32	2.26	2.22	2.18
Xanthurenic acid	2.40	2.30	2.24	2.20	2.17
Glucose	2.26	2.12	2.06	2.03	1.99
Glycochenodeoxycholate	2.23	2.24	2.21	2.18	2.14
Xanthine	2.15	2.00	1.95	1.91	1.89
Glycerol‐3‐phosphate	2.05	1.93	1.92	1.88	1.85
Taurine	1.84	1.74	1.70	1.69	1.68
Oxalic acid	1.80	1.70	1.71	1.69	1.66
D‐Leucic acid	1.74	1.62	1.60	1.58	1.55
Lactate	1.65	1.57	1.60	1.58	1.56
Hypoxanthine	1.63	1.58	1.55	1.52	1.53
Pyruvate	1.51	1.52	1.55	1.52	1.50
Alanine	1.43	1.33	1.29	1.28	1.27
3‐Indoxyl sulfate	1.42	1.35	1.34	1.33	1.33
1‐Methylguanosine	1.39	1.30	1.27	1.31	1.28
Sorbitol	1.39	1.51	1.48	1.46	1.45
3‐methyl‐2‐oxovaleric acid	1.38	1.29	1.28	1.26	1.24
Propionate	1.33	1.25	1.26	1.24	1.22
Indole‐3‐lactate	1.29	1.21	1.20	1.18	1.17
Reduced glutathione	1.28	1.21	1.18	1.16	1.18
Lactose	1.21	1.19	1.16	1.14	1.14
L‐kynurenine	1.16	1.08	1.16	1.14	1.12
Adenosine Monophosphate	1.12	1.07	1.08	1.09	1.08

*Note*: Variable importance in projection (VIP) component scores from partial least squares discriminant analysis (PLS‐DA). Scores ≥1.0 indicate a significant contribution to differences among time points.

**FIGURE 6:**
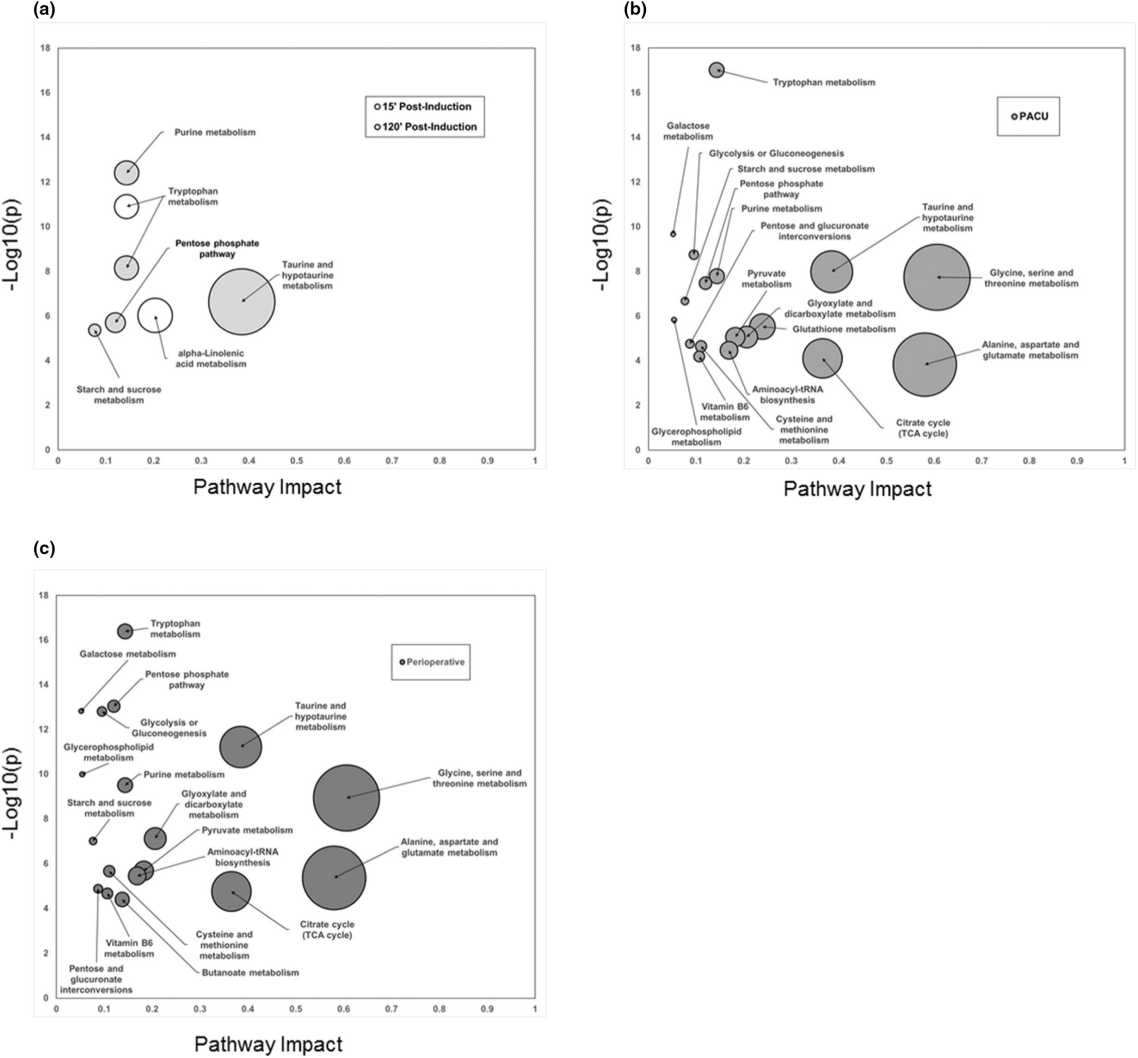
Perioperative metabolite pathway dynamics. Tryptophan and linolenic acid metabolism increase following induction (a, white). Carbohydrate and nucleotide metabolite networks are prominent after sustained surgical stimulation (a, light gray). In the post‐anesthesia care unit (PACU), amino acid catabolism and gluconeogenesis dominate the metabolome (b). Analyses of the metabolomic network over the whole perioperative period show broad amino acid and carbohydrate catabolism (c). Pathway impact derived from relative betweenness centrality of the metabolic networks and significance of each pathway determined by enrichment analyses (presented as negative log of the raw p‐value). Data presented have pathway impact >0.05 and false detection rate corrected *p*‐values <0.05.

Metabolomic pathway and network analyses show the dynamic changes in metabolic programs over the course of the perioperative course (Figure 6). After induction and intubation, changes in tryptophan and alpha‐linolenic acid metabolism are prevalent in the metabolite profiles (Figure 6a). Two hours after induction and after more than an hour of surgical stimulus, signs of glycogenolysis, pentose phosphate shunting, and nucleic acid metabolism appear alongside increases in bile acid synthesis, rising taurine concentrations, and decreasing tryptophan levels (Figure 6a). Following emergence and 1 h in the recovery room, gluconeogenic pathways associated with amino acid catabolism and glycolytic pathways associated with increased TCA cycle intermediates are highly prevalent in the metabolome with smaller contributions from glycerolipid and vitamin metabolism (Figure 6b). These pathways dominate the perioperative metabolomic network (Figure 6c).

## 4 DISCUSSION

Energy metabolism is altered by both volatile anesthetics and surgical stress in the perioperative period. Our data show that volatile anesthetics impair basal insulin secretion, uncoupling glucose and insulin homeostasis. Subsequent surgical stresses led to hyperglycemia and eventual recovery of insulin secretion and normalized insulin‐to‐glucose ratios in later stages of the procedure and in recovery. Metabolite data showed a prominent role for glycogenolysis, the pentose phosphate pathway, and selective protein metabolism during the early phase of the anesthetic and surgical procedure. Glycolytic metabolites and evidence of gluconeogenesis did not appear until patients were in recovery. These data show that energy metabolism with a volatile anesthetic relies on non‐glycolytic pathways likely due to the suppression of insulin secretion. Lack of insulin signaling decreases energy production through glycolysis (e.g., from reduced insulin stimulated dephosphorylation of phosphofructokinase) necessitating generation of ATP through glycogen metabolism and the pentose phosphate shunt. The observed hyperglycemia results from a combination of reduced insulin‐mediated glycolysis and of glycogenolysis early in the anesthetic and surgical procedure. Characteristic features of the neuroendocrine stress response were apparent in recovery (e.g., gluconeogenesis and protein catabolism). We did not find strong evidence for lipid oxidation or insulin resistance during the anesthetic or in recovery.

In the present study, the main effect of volatile anesthetics is a suppression of basal insulin secretion prior to the start of the surgical procedure. These data are consistent with preclinical experiments in canine and porcine models which found that volatile anesthetics briskly suppress insulin secretion in the absence of surgical stimulus (Horber et al., 1990; Saho et al., 1997; Vore et al., 2001). A proposed mechanism for this effect is inhibiting closure of K_ATP_ in β‐cells, attenuating release of insulin granules (Desborough et al., 1993; Gingerich et al., 1974; Tanaka et al., 2009). Two small clinical studies previously concluded that volatile anesthetics reduce insulin secretion and impair glucose tolerance using modified intravenous glucose tolerance tests (IVGTT) (Diltoer & Camu, 1988; Tanaka et al., 2005). When compared to the non‐anesthetized state, subjects had reduced insulin levels and slower glucose disappearance rates during IVGTT under volatile anesthetic without a surgical stimulus. Our data support the conclusion that decreased insulin levels result from suppressed insulin secretion mediated by the volatile anesthetic at the level of the beta‐cell. We extend these previous data by showing this is not a function of altered hepatic insulin extraction and the circulating metabolome is consistent with energy generation through non‐glycolytic pathways due to reduced insulin secretion and signaling.

Very few amino acids changed during the early phase of anesthesia and surgery in this study, but protein catabolism and gluconeogenesis robustly contributed to post‐operative hyperglycemia. These data are consistent with previous studies showing negative nitrogen balance following anesthesia and surgery. Carli et al. used isotope labeled leucine and glucose tracers to examine protein and glucose metabolism during surgery with a volatile anesthetic (Carli et al., 1990; Schricker et al., 2001; Vore et al., 2001). They found decreased leucine metabolism, decreased protein synthesis, and reduced glucose clearance, all of which normalized in recovery. These are all elegant, well‐conducted, technically demanding studies, but their interpretation is limited by the sample sizes and the complexity of energy metabolism. While the glucose kinetics are relatively simple to interpret, leucine kinetics are not representative of whole‐body protein metabolism. In our study, leucine concentrations remain stable but leucic acid levels, a leucine metabolite, rise from baseline through recovery suggesting increased leucine appearance balancing the increased catabolism. Recent examination of the amino acid dynamics in human starvation using metabolomics call into question many of the underlying assumptions of stable‐isotope techniques (Steinhauser et al., 2018). Differential release and use of amino acids appear to control the initiation and maintenance of lipid metabolism during prolonged fasting. Our data are consistent with these findings as we found evidence of selective amino acid metabolism early in the anesthetic and surgery followed by more widespread amino acid metabolism along gluconeogenic pathways in recovery.

We observed several previously undescribed alterations in perioperative metabolism of tryptophan, glutamate, and taurine, each of which plays a role in energy metabolism, inflammation, and neurotransmission. The most striking of these was the profound decrease in tryptophan levels, which was more drastic and rapid than seen in simple starvation (Steinhauser et al., 2018). Tryptophan is an essential amino acid that serves as a gluconeogenic substrate and the precursor for serotonin, tryptamine, and kynurenine synthesis. This profound decrease is intriguing and potentially important as tryptophan depletion can alter the mood and affect as a result of altered serotonin concentrations (Park et al., 1994; van der Veen et al., 2007; Young, 2013). Taurine is a highly abundant non‐protein amino acid that has multiple important roles including bile acid synthesis, reduction of oxidative stress, and neurotransmission. Myriad studies of taurine supplementation have ascribed wide‐ranging functions including modulation of insulin secretion and attenuation of responses to cortisol (Mezzomo et al., 2019; Santos‐Silva et al., 2015). Our data showed an elevation of circulating taurine levels, most likely from reduced incorporation into bile acids. Finally, glutamate is the most common excitatory neurotransmitter in vertebrates, agonizes a large family of cellular receptors, and serves as a precursor for gamma‐aminobutyric acid (GABA) synthesis (Magi et al., 2019). In addition, glutamate is a non‐essential amino acid used in protein synthesis and a key anapleurotic intermediate in the TCA cycle. In the central nervous system, the role of glutamate as a neurotransmitter cannot be easily separated from its role in cellular energy metabolism. The reduced circulating glutamate concentrations in the recovery period in our subjects likely results from direct contributions to energy metabolism as glutamate may be a preferred substrate during energy‐depleted conditions.

We found very little evidence of lipid metabolism during anesthesia and surgery. The increases in circulating linoleic and linolenic acid and their metabolites immediately following induction is most likely from the propofol lipid emulsion, which is made from soybean oil, egg lecithin (glycerophospholipids), and glycerol. Soybean oils are predominantly composed of linoleic and linoleic acid in addition to smaller amounts of other fatty acid species (Baker & Naguib, 2005). We found increased glycerolipid metabolism after emergence and 1 hour in recovery. It should be noted that we did not comprehensively measure the lipidome. These results are consistent with recent evidence that endogenous lipid mobilization does not occur until more than 24 h of fasting in healthy human subjects (Steinhauser et al., 2018). The widespread use of propofol‐based anesthetics highlights the need for understanding how the lipid carrier may alter intraoperative and post‐operative metabolic function.

### 4.1 Limitations

This study has several important limitations. This was an observational study of the response to volatile anesthetic agents and we did not compare responses to other anesthetic regimens. The procedures our subjects underwent were similar in intensity and invasiveness and the 30–60 min between intubation and surgical incision provided a stimulus‐free period for evaluating the effect of the volatile anesthetic, but these conditions limit the generalizability of the results. Other procedures with different temporal characteristics, intensities, durations, or anesthetic medications may elicit different glucose and insulin profiles. Notably, use of an epidural or a combination of preoperative amino acid supplementation with remifentanil/sevoflurane anesthesia may blunt the elaboration of cortisol and the catabolic effects of the neuroendocrine stress response (Fukuta et al., 2020; Lattermann et al., 2002). Seven subjects had procedures beginning around midday while nine had first procedures. Cortisol levels are affected by circadian rhythms even while fasting, but we did not observe differences between these groups at baseline. Two subjects were fed in PACU prior to the final blood sampling. One of these was not included in the metabolomics analyses due to transfusion of blood bank erythrocytes, which contain glucose among other preservatives and anticoagulants. Hormone and metabolite data from these subjects were not outside the ranges of the other subjects. Overall, we do not see a major effect of these two data points on the study outcomes. There was also some heterogeneity in the pre‐operative medication subjects received, the majority of which do not affect glucose metabolism, and the induction regimens were nearly identical. Opioids can blunt cortisol release at both the pituitary and adrenal glands and may have contributed low cortisol levels following intubation. It is important to note that dexamethasone (given to 11 subjects) is not associated with perioperative hyperglycemia (Murphy et al., 2014).

### 4.2 Conclusions and future directions

Surgical and anesthetic interventions induce a wide range of metabolic changes which may affect the response to and recovery from the controlled traumas of surgery. Our results highlight the complexity of energy metabolism and the competing effects of anesthetic medications and the surgical stress response. A careful examination of the interactions among stress metabolism, anesthetic medications, and patient metabolic dysfunction is needed in order to provide a rational foundation for design of perioperative metabolic care.

## CONFLICT OF INTEREST STATEMENT

No authors have conflicts of interest to declare related to this study.

## ETHICS STATEMENT

This study was approved by the University of Vermont Institutional Review Board (protocol M14‐291). All subjects gave written informed constent prior to participation in the study.

## Supporting information


Data S1.
