# Supplementary material for: The neuroendocrine stress response compensates for suppression of insulin secretion by volatile anesthetic agents: An observational study
Source: Physiol Rep. 2023 Feb 17;11(4):e15603. doi: 10.14814/phy2.15603 (PMC9937792; doi:10.14814/phy2.15603)
Supplement: Supplementary file 1 — Data S1. [file PHY2-11-e15603-s001.docx]

**Metabolism During Surgery with Volatile Anesthetic - Supplementary Data**

**
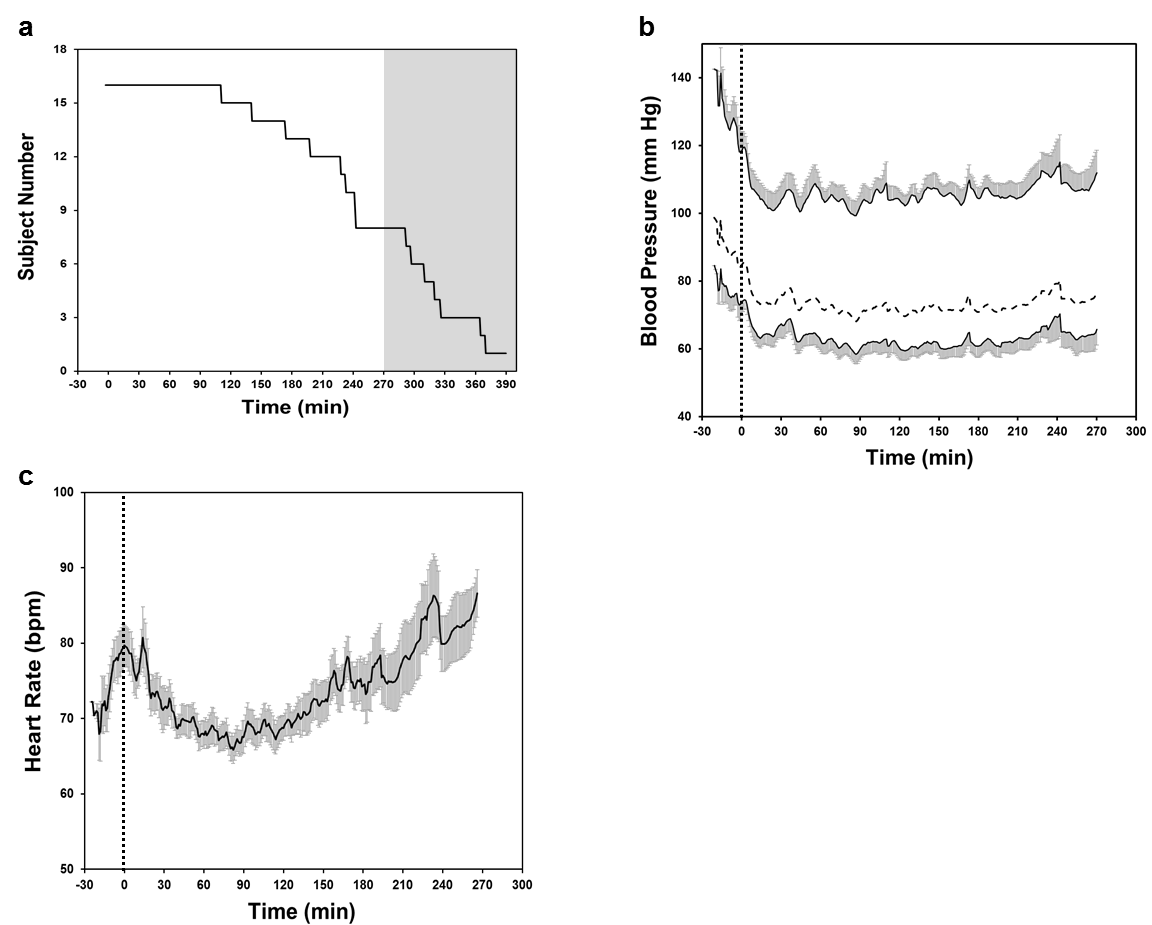
Supplementary Figure 1**

**Supplementary Figure 1 –** **Procedure Duration and Hemodynamics.** Kaplan-Meier time to event curve for anesthetic and surgical duration (a). Shaded area shows region censored in all subsequent figures. Mean arterial (dashed line), systolic (upper solid line), diastolic (lower solid line) blood pressures prior to induction and during the procedure (b). Heart rate (beats per minute) prior to induction and during the procedure (c). Induction is noted at time 0 minutes with a vertical dotted line. Hemodynamic data underwent 5 minute moving average smoothing and are presented as mean ± SEM.

**Supplementary Figure 2**

**
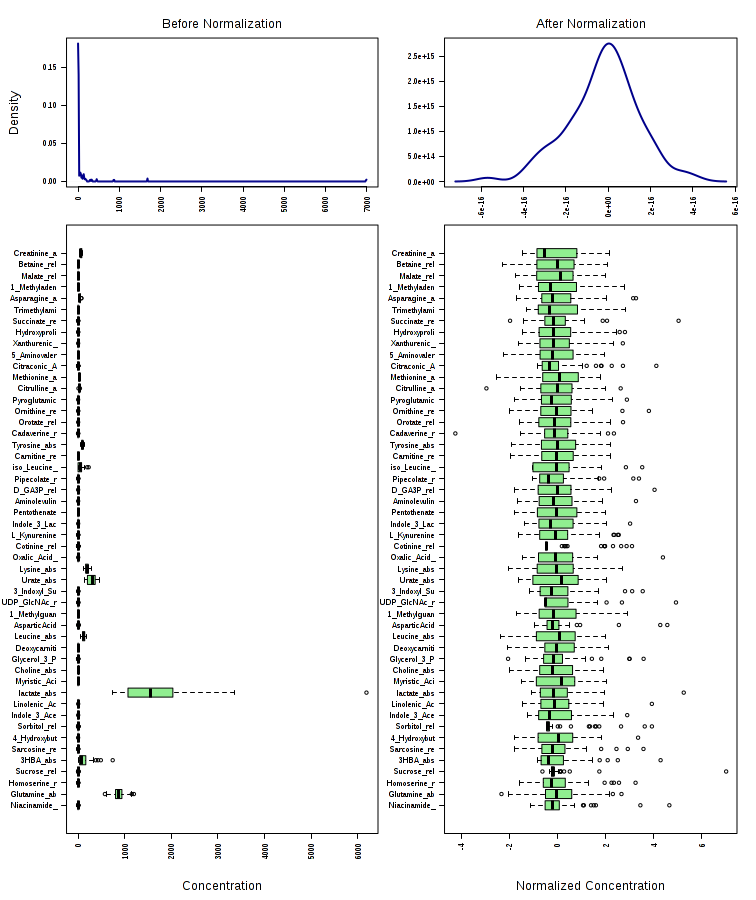
**

**Supplementary Figure 2 – Metabolomics Data Normalization.** Panels on the left show the concentrations and density plot for the raw metabolomics data. Panels on the right show the normalized concentrations and density plot after auto-scaling. Data were mean-centered and normalized to their standard deviation. Any analytes with >50% missing values were removed. Analytes with <50% missing values had any missing values replaced with half of the minimum detected value.

**Supplementary Table 1 – Metabolite one-way ANOVA**

| **Metabolite** | **f value** | **p value** | **-log10(p)** | **FDR** | **Tukey's HSD** |
| --- | --- | --- | --- | --- | --- |
| Tryptophan | 20.51 | 0.00 | 8.18 | 0.00 | 2-1; 3-1; 4-1; 4-2 |
| Xanthurenic Acid | 15.13 | 0.00 | 6.48 | 0.00 | 2-1; 3-1; 4-1 |
| Glyceraldehyde | 13.88 | 0.00 | 6.04 | 0.00 | 3-1; 4-1; 4-2; 4-3 |
| Glycochenodeoxycholate | 13.77 | 0.00 | 6.00 | 0.00 | 2-1; 3-1; 4-1 |
| Uridine | 10.84 | 0.00 | 4.91 | 0.00 | 2-1; 3-1; 4-1; 4-2; 4-3 |
| Glucose | 10.43 | 0.00 | 4.75 | 0.00 | 3-1; 4-1; 4-2; 4-3 |
| Glycerol-3-Phosphate | 10.29 | 0.00 | 4.70 | 0.00 | 4-1; 3-2; 4-2; 4-3 |
| 13-HODE | 9.20 | 0.00 | 4.26 | 0.00 | 2-1; 3-2; 4-2 |
| Xanthine | 8.05 | 0.00 | 3.78 | 0.00 | 3-1; 4-1; 4-2 |
| Taurine | 7.50 | 0.00 | 3.54 | 0.00 | 3-1; 4-1; 3-2; 4-2 |
| Linolenic Acid | 5.60 | 0.00 | 2.68 | 0.02 | 2-1; 3-2; 4-2 |
| Propionate | 5.38 | 0.00 | 2.58 | 0.02 | 4-1; 4-2; 4-3 |
| Oxalic Acid | 5.11 | 0.00 | 2.45 | 0.03 | 3-1; 4-1; 4-2 |
| D-Leucic Acid | 4.64 | 0.01 | 2.22 | 0.05 | 4-1; 4-2 |
| Glutamicacid | 4.58 | 0.01 | 2.19 | 0.05 | 4-2; 4-3 |
| Pyruvate | 4.50 | 0.01 | 2.15 | 0.05 | 4-1; 4-2; 4-3 |
| Aminolevulinate | 4.33 | 0.01 | 2.07 | 0.05 | 3-1; 4-3 |
| lactate | 3.96 | 0.01 | 1.89 | 0.08 | 3-1; 4-1; 4-2 |
| Hypoxanthine | 3.84 | 0.01 | 1.83 | 0.08 | 4-1; 4-2 |
| Isoleucine | 3.54 | 0.02 | 1.68 | 0.11 | 2-1; 3-2; 4-2 |
| 1-Methylguanosine | 3.40 | 0.02 | 1.61 | 0.12 | 2-1; 3-1; 4-1 |
| Sorbitol | 3.35 | 0.03 | 1.59 | 0.13 | 3-1; 4-1; 3-2; 4-2 |
| Oxalacetate | 3.28 | 0.03 | 1.55 | 0.13 | 4-1; 4-2; 4-3 |
| Ribose-5-Phosphate | 3.27 | 0.03 | 1.55 | 0.13 | 3-1; 3-2 |
| 3-Indoxyl Sulfate | 3.18 | 0.03 | 1.50 | 0.13 | 3-1; 4-1 |
| Reduced glutathione | 3.09 | 0.03 | 1.46 | 0.14 | 4-1; 4-2; 4-3 |
| 3-Methy-2-Oxovaleric Acid | 3.08 | 0.04 | 1.45 | 0.14 | 4-1; 4-2 |
| Phenylpyruvic Acid | 2.94 | 0.04 | 1.38 | 0.16 | 2-1; 3-1 |
| L-Kynurenine | 2.89 | 0.04 | 1.36 | 0.16 | 4-1; 4-2; 4-3 |
| Alanine | 2.87 | 0.05 | 1.35 | 0.16 | 4-1; 4-2 |
| Lactose | 2.66 | 0.06 | 1.24 | 0.19 | 4-1; 4-2 |
| Linoleic Acid | 2.66 | 0.06 | 1.24 | 0.19 | 2-1 |
| **Abbreviations:** false discovery rate corrected p-value, FDR; honest significant difference; HSD. | | | | | |

**Supplementary Table 2 – Metabolite VIP Component Scores**

|  | **Component VIP Score** | | | | |
| --- | --- | --- | --- | --- | --- |
| **Metabolite** | **1** | **2** | **3** | **4** | **5** |
| Tryptophan | 2.66 | 2.56 | 2.49 | 2.45 | 2.40 |
| Glyceraldehyde | 2.49 | 2.32 | 2.26 | 2.22 | 2.18 |
| Xanthurenic Acid | 2.40 | 2.30 | 2.24 | 2.20 | 2.17 |
| Glucose | 2.26 | 2.12 | 2.06 | 2.03 | 1.99 |
| Glycochenodeoxycholate | 2.23 | 2.24 | 2.21 | 2.18 | 2.14 |
| Xanthine | 2.15 | 2.00 | 1.95 | 1.91 | 1.89 |
| Glycerol-3-Phosphate | 2.05 | 1.93 | 1.92 | 1.88 | 1.85 |
| Taurine | 1.84 | 1.74 | 1.70 | 1.69 | 1.68 |
| Oxalic Acid | 1.80 | 1.70 | 1.71 | 1.69 | 1.66 |
| D-Leucic Acid | 1.74 | 1.62 | 1.60 | 1.58 | 1.55 |
| Lactate | 1.65 | 1.57 | 1.60 | 1.58 | 1.56 |
| Hypoxanthine | 1.63 | 1.58 | 1.55 | 1.52 | 1.53 |
| Pyruvate | 1.51 | 1.52 | 1.55 | 1.52 | 1.50 |
| Alanine | 1.43 | 1.33 | 1.29 | 1.28 | 1.27 |
| 3-Indoxyl Sulfate | 1.42 | 1.35 | 1.34 | 1.33 | 1.33 |
| 1-Methylguanosine | 1.39 | 1.30 | 1.27 | 1.31 | 1.28 |
| Sorbitol | 1.39 | 1.51 | 1.48 | 1.46 | 1.45 |
| 3-Methyl-2-Oxovaleric Acid | 1.38 | 1.29 | 1.28 | 1.26 | 1.24 |
| Propionate | 1.33 | 1.25 | 1.26 | 1.24 | 1.22 |
| Indole-3-Lactate | 1.29 | 1.21 | 1.20 | 1.18 | 1.17 |
| Reduced Glutathione | 1.28 | 1.21 | 1.18 | 1.16 | 1.18 |
| Lactose | 1.21 | 1.19 | 1.16 | 1.14 | 1.14 |
| L-Kynurenine | 1.16 | 1.08 | 1.16 | 1.14 | 1.12 |
| Adenosine Monophosphate | 1.12 | 1.07 | 1.08 | 1.09 | 1.08 |
| Ribose 5 P | 1.09 | 1.02 | 0.99 | 1.00 | 1.01 |
| 1-Methyladenosine | 1.07 | 1.01 | 1.00 | 0.99 | 0.98 |
| Choline | 1.05 | 0.98 | 0.96 | 0.97 | 0.95 |
| Citrulline | 1.03 | 0.97 | 0.95 | 1.00 | 1.00 |
| Oxalacetate | 1.02 | 1.02 | 0.99 | 0.97 | 0.99 |
| 3-Hydroxybutyric Acid | 1.02 | 0.95 | 1.04 | 1.02 | 1.03 |
| Trimethylamine N oxide | 0.97 | 0.91 | 0.89 | 0.90 | 0.90 |
| N2,N2 Dimethylguanosine | 0.94 | 0.92 | 0.94 | 0.98 | 0.98 |
| Indole 3 Acetic Acid | 0.92 | 0.96 | 0.97 | 0.95 | 0.94 |
| Glutamicacid | 0.92 | 0.95 | 0.93 | 0.92 | 0.90 |
| 2 Hydroxyglutarate | 0.90 | 0.86 | 0.84 | 0.87 | 0.86 |
| Arachidonate | 0.88 | 1.03 | 1.01 | 1.02 | 1.04 |
| Margaric Acid | 0.88 | 0.84 | 0.81 | 0.80 | 0.78 |
| 5 Aminovaleric Acid | 0.86 | 0.85 | 0.89 | 0.94 | 0.92 |
| Leucine | 0.86 | 0.82 | 0.91 | 0.90 | 0.92 |
| D GA3P | 0.86 | 0.83 | 0.82 | 0.83 | 0.83 |
| Hydroxyproline | 0.85 | 0.79 | 0.79 | 0.91 | 0.90 |
| Deoxycarnitine | 0.85 | 0.80 | 0.78 | 0.83 | 0.83 |
| Inositol | 0.81 | 0.95 | 0.95 | 0.96 | 0.95 |
| Tyrosine | 0.80 | 0.81 | 0.79 | 0.81 | 0.80 |
| Myristic Acid | 0.80 | 0.94 | 0.93 | 0.92 | 0.90 |
| Uridine | 0.79 | 0.75 | 0.73 | 0.74 | 0.73 |
| Threonine | 0.77 | 0.72 | 0.74 | 0.72 | 0.73 |
| Pipecolate | 0.75 | 0.74 | 0.75 | 0.75 | 0.76 |
| Betaine | 0.75 | 0.75 | 0.74 | 0.74 | 0.79 |
| Dimethylglycine | 0.75 | 0.72 | 0.81 | 0.82 | 0.84 |
| Creatine | 0.74 | 0.88 | 0.88 | 0.87 | 0.90 |
| Cadaverine | 0.74 | 0.71 | 0.69 | 0.68 | 0.67 |
| Alpha Ketoglutaric Acid | 0.73 | 1.04 | 1.01 | 1.03 | 1.06 |
| Asparagine | 0.73 | 0.77 | 0.75 | 0.74 | 0.81 |
| isoValeric Acid | 0.72 | 0.69 | 0.69 | 0.68 | 0.72 |
| Carnitine | 0.69 | 0.80 | 0.79 | 0.81 | 0.85 |
| Glycine | 0.69 | 0.78 | 0.77 | 0.77 | 0.77 |
| Guanidinoacetate | 0.68 | 0.65 | 0.64 | 0.67 | 0.67 |
| Sucrose | 0.67 | 0.63 | 0.61 | 0.60 | 0.60 |
| PPA | 0.66 | 0.71 | 0.69 | 0.68 | 0.72 |
| Proline | 0.64 | 0.60 | 0.59 | 0.58 | 0.58 |
| Ornithine | 0.62 | 0.59 | 0.57 | 0.58 | 0.74 |
| Linoleic Acid | 0.62 | 0.62 | 0.60 | 0.62 | 0.61 |
| Pyroglutamic Acid | 0.61 | 0.58 | 0.73 | 0.72 | 0.70 |
| Serine | 0.61 | 0.61 | 0.60 | 0.59 | 0.59 |
| Homoserine | 0.60 | 0.56 | 0.61 | 0.61 | 0.62 |
| UDP GlcNAc | 0.58 | 0.58 | 0.61 | 0.67 | 0.71 |
| Allantoin | 0.57 | 0.56 | 0.64 | 0.64 | 0.63 |
| Acetylcarnitine | 0.57 | 0.68 | 0.70 | 0.71 | 0.74 |
| Glutamine | 0.56 | 1.04 | 1.01 | 1.01 | 1.03 |
| 1 3 Methylhistidine | 0.54 | 0.57 | 0.57 | 0.56 | 0.57 |
| 2 Aminoadipate | 0.51 | 0.63 | 0.65 | 0.65 | 0.64 |
| Oxidized glutathione | 0.50 | 0.46 | 0.49 | 0.53 | 0.53 |
| Orotate | 0.48 | 0.81 | 0.89 | 0.89 | 0.88 |
| Glucoronate | 0.47 | 0.72 | 0.77 | 0.75 | 0.75 |
| Kynurenic Acid | 0.45 | 0.42 | 0.43 | 0.48 | 0.48 |
| Citraconic Acid | 0.45 | 0.43 | 0.43 | 0.43 | 0.53 |
| Urate | 0.45 | 0.59 | 0.71 | 0.71 | 0.70 |
| iso Leucine | 0.42 | 0.42 | 0.42 | 0.43 | 0.45 |
| Malate | 0.40 | 0.38 | 0.42 | 0.43 | 0.44 |
| Fructose | 0.34 | 0.63 | 0.64 | 0.63 | 0.65 |
| Cystine | 0.34 | 0.51 | 0.50 | 0.50 | 0.50 |
| Glycerate | 0.34 | 0.33 | 0.58 | 0.57 | 0.57 |
| Sarcosine | 0.33 | 0.32 | 0.31 | 0.31 | 0.31 |
| Linolenic Acid | 0.31 | 0.57 | 0.57 | 0.59 | 0.59 |
| Phenylalanine | 0.29 | 0.56 | 0.59 | 0.59 | 0.62 |
| N Acetylneuraminate | 0.28 | 0.55 | 0.62 | 0.69 | 0.71 |
| 4 Pyridoxic acid | 0.23 | 0.30 | 0.46 | 0.47 | 0.46 |
| Pentothenate | 0.22 | 0.61 | 0.61 | 0.61 | 0.61 |
| AsparticAcid | 0.21 | 0.77 | 0.77 | 0.77 | 0.77 |
| Methionine | 0.19 | 0.17 | 0.19 | 0.28 | 0.38 |
| N AcetylGlycine | 0.18 | 0.48 | 0.47 | 0.46 | 0.46 |
| Succinate | 0.16 | 0.42 | 0.52 | 0.57 | 0.64 |
| Oxypurinol | 0.14 | 0.39 | 0.43 | 0.43 | 0.56 |
| Hippuric Acid | 0.14 | 0.28 | 0.27 | 0.43 | 0.49 |
| Adipic Acid | 0.12 | 0.75 | 0.75 | 0.75 | 0.78 |
| Lysine | 0.09 | 0.22 | 0.26 | 0.40 | 0.43 |
| 13 HODE | 0.08 | 0.24 | 0.36 | 0.43 | 0.51 |
| Cotinine | 0.08 | 0.56 | 0.54 | 0.67 | 0.67 |
| 2 Hydroxyisovaleric Acid | 0.07 | 0.11 | 0.31 | 0.30 | 0.32 |
| Histidine | 0.06 | 0.60 | 0.60 | 0.70 | 0.70 |
| Creatinine | 0.06 | 0.08 | 0.64 | 0.64 | 0.63 |
| 4 Hydroxybutyrate | 0.05 | 0.50 | 0.49 | 0.49 | 0.57 |
| Niacinamide | 0.04 | 0.34 | 0.33 | 0.33 | 0.38 |
| Valine | 0.02 | 0.04 | 0.26 | 0.40 | 0.40 |
| Aminolevulinate | 0.02 | 0.16 | 0.25 | 0.56 | 0.57 |
| Arginine | 0.00 | 0.40 | 0.41 | 0.44 | 0.59 |
| **Abbreviations:** variable importance in projection, VIP. | | | | | |
